# Primary mental health care for adults with mild intellectual disabilities: a focus group study of care professionals’ perspectives

**DOI:** 10.3399/BJGPO.2023.0247

**Published:** 2024-10-30

**Authors:** Katrien PM Pouls, Mathilde Mastebroek, Suzanne A Ligthart, Willem JJ Assendelft, Monique CJ Koks-Leensen, Geraline L Leusink

**Affiliations:** 1 Department of Primary and Community Care, Radboud University Medical Center, Nijmegen, The Netherlands

**Keywords:** mental health, intellectual disability, primary healthcare, general practitioners

## Abstract

**Background:**

GPs and mental health nurse practitioners (MHNPs) often feel ill equipped to provide mental health (MH) care to people with mild intellectual disabilities (MID). This is worrying, as insufficient primary MH care may lead to more severe or chronic problems. To improve primary MH care for this patient group, account must be taken of the experiences and needs of GPs and MHNPs providing the care.

**Aim:**

To explore GPs’ and MHNPs’ experiences, needs, and recommendations for improvement regarding primary MH care for adults with MID.

**Design & setting:**

A qualitative study was undertaken using focus groups with GPs and MHNPs in the Netherlands.

**Method:**

The focus groups were guided by topics based on an interview study with adults with MID receiving primary MH care. Transcripts were analysed by thematic analysis.

**Results:**

Four focus groups, with 19 GPs and nine MHNPs, revealed four themes describing the needs and perceived complexity involved in providing MH care to patients with both MID and MH problems: (1) GPs’ and MHNPs’ struggles with adapting to challenging patient characteristics; (2) importance and difficulties of establishing a good doctor–patient relationship; (3) facilitating and hampering roles of the patient’s network; and (4) GPs’ and MHNPs’ challenges to provide care in the healthcare chain.

**Conclusion:**

GPs and MHNPs often experience providing care and support to this patient group as burdensome. It is important to consider the MID throughout the MH trajectory, to invest in a strong doctor–patient relationship, and to establish a stable, sustainable network and coordinated collaborative care around the patient.

## How this fits in

Mental health (MH) problems are highly prevalent in people with mild intellectual disabilities (MID), who have a high use of primary care services compared with people with no MID, or with MID without MH problems. However, GPs and mental health nurse practitioners (MHNPs) do not always feel equipped to provide MH care to this patient group, although they acknowledge that they have an important role in the detection, treatment, and follow-up of MH problems. In order to improve primary MH care for this patient group, it is essential to take into account the — hitherto unincluded — experiences and needs of care professionals who provide the care. This study explores GPs’ and MHNPs’ reflections regarding primary MH care for adults with MID, including practice-based insights that may contribute positively to the quality of care.

## Introduction

In many countries, the GP is the first point of contact for people with MH problems, and the gatekeeper to MH services. However, GPs do not always feel equipped to provide MH care to people with MID,^1,2^ which is defined as a significant deficit in intellectual (intelligence quotient [IQ] range 50–85) and adaptive functioning.^3^ This is worrying, as insufficient primary MH care in this group may lead to more severe or chronic MH problems.^4^

People with both MID and MH problems put a high demand on primary care practices.^5^ The prevalence of MH problems in people with MID is twice that of the general population estimate.^6^ Furthermore, this patient group consults their GP more frequently compared with patients without intellectual disabilities (ID) or with MID alone.^5^

Previous research indicates that current primary MH care for people with MID requires improvement.^2^ GPs internationally lack evidence-based knowledge and MH screening tools for people with MID,^1,7^ struggle to identify and register the MID, and therefore have difficulty taking the MID into account when establishing the correct MH diagnosis and treatment.^2^ This may lead to underdiagnosis, overmedication, and the progression of MH problems.^2,7^

In order to improve primary MH care, it is important to stay close to the actual experiences of patients to whom this care is provided and of care providers who provide the care. In The Netherlands, this is the GP, often supported by a MHNP. An MHNP works under GP supervision, has a role in assessing MH symptoms, and gives short periods of treatment and support to people with mild MH problems.

Results of an interview study revealed that people with both MID and MH problems have various additional needs and expectations regarding their GP, family, and professional carers, but their need for self-determination remains important. This stems from a sense of vulnerability when visiting their GP, which is related to their MID and reinforced by MH problems.^8^ It is known from previous studies that GPs experience difficulties in providing care to patients with solely MID or MH problems,^9–12^ leading to the assumption that the combination of MID and MH problems entails even greater or additional difficulties for the GP. We found only one study — exploring the experiences of Norwegian GPs providing care to people with intellectual disability (ID) — with a special focus on combined ID and MH problems. That study’s participants, who had above average experience with people with ID, felt insecure in treating these patients and experienced problems in referring patients when problems became too complex for primary care.^1^

Further knowledge that could guide quality of care improvement is lacking, given GPs’ and MHNPs’ specific needs regarding primary MH care for people with MID. Therefore, the aim of this qualitative study is to explore GPs’ and MHNPs’ experiences, needs, and recommendations for improvement regarding primary MH care for adults with MID.

## Method

### Setting and sample

To broadly explore GPs’ and MHNPs’ perspectives on the care provided to patients with MID and MH problems, we conducted a focus group study. We recruited GPs and MHNPs in several Dutch regions in November and December 2022. Our recruitment strategy entailed contacting our personal networks through local GP networks, national GP and MHNP organisations, and LinkedIn. To ensure distribution across The Netherlands, we applied purposive sampling. Varying levels of experience with this patient group were included.

Candidates received study information by email, and written informed consent was obtained. The target number of participants in each focus group was 5–10, and focus groups were conducted at a location of the groups’ choice, including online. All participants were offered a €25 (approximately 21 GBP) gift voucher and postgraduate education accreditation points.

### Data collection

The focus groups were guided by a topic guide based on four themes emerging from a previous interview study among patients with MID who received primary MH care (see supplementary information).^8^ This topic guide was critically scrutinised by both the research team and the study’s advisory group, the latter consisting of representatives from primary care, ID care, MH care, addiction care, and associations for patients with ID. This was done to ensure that GPs’ and MHNPs’ perspectives were collected as completely as possible.

Before the focus group meetings, the following participant basic characteristics were ascertained: sex; age; number of years of work experience; and self-perceived level of experience with patients with MID. All focus groups were moderated by the same researcher (KP), who is an ID physician, assisted by a second researcher, either one of two senior researchers (MK, MM) or a PhD candidate (AB).

### Analysis

The focus group discussions were audio-recorded and transcribed verbatim. The transcripts were analysed following the principles of thematic analysis,^13^ supported by ATLAS.ti software (version 22.0.11). Coding was conducted by two researchers independently (KP and MK or MM). Codes were then collated in preliminary themes and repeatedly discussed among the research team (KP, MK, MM, SL, WA, GL) to refine and define the final themes. The Consolidated Criteria for Reporting Qualitative research (COREQ) criteria list was used to guide the analysis and the report.^14^

## Results

Nineteen GPs and nine MHNPs participated in four focus groups held in February and March 2023. Their age ranged from 29–65 years; 20 were female. The median work experience was 8 years (range 1–28 years), and most participants reported low to average experience with people with MID in their practice (Table 1). One focus group took place online, the others in person.

**Table 1.** Participants’ characteristics

Focus group number^a^	Age, years	Sex	GP or MHNP	Work experience, years	Experience with patients with MID
1	36	F	MHNP	7	++
1	46	F	GP	16	++
1	63	F	MHNP	5	++
1	46	F	GP	15	+
1	65	M	MHNP	10	++
1	46	M	GP	15	++
1	49	F	GP	14	++
2	39	F	GP	1	+++
2	37	F	GP	5	+
2	35	F	GP	4	++
2	36	F	GP	3	++
2	40	F	GP	5	++
3	62	F	MHNP	18	++
3	36	F	MHNP	3	+++
3	52	F	MHNP	11	+
3	56	F	MHNP	8	+++
3	58	F	MHNP	8	+
3	55	F	MHNP	9	+
4	45	F	GP	21	+
4	32	M	GP	3	++
4	45	F	GP	17	++
4	31	M	GP	2	+
4	56	M	GP	22	++
4	53	M	GP	20	++
4	34	M	GP	7	+
4	58	F	GP	28	+
4	34	F	GP	5	+
4	29	M	GP	1	+

a Focus groups 1, 2, 4 were held in person, focus group 3 was held online. + = low, ++ = average, +++ = above average of experience with patients with MID.^.^

F = female. M = male. MHNP = mental health nurse practitioner

Overall, the participants perceived their patients with both MID and MH problems as complex patients who, given their high care needs, put pressure on their general practices. Participants felt responsible for this patient group but found it difficult to provide them with the appropriate (MH) care, leading to participants feeling frustration, uncertainty, and despondency. Reasons for the perceived complexity in delivering (MH) care to patients with both MID and MH problems were captured in the following four main themes emerging from the focus groups: (1) GPs’ and MHNPs’ struggles with adapting to challenging patient characteristics; (2) importance and difficulties of establishing a good doctor–patient relationship; (3) facilitating and hampering roles of the patient’s network; (4) GPs’ and MHNPs’ challenges to provide care in the healthcare chain (Table 2). Below, each theme is described, ending in each case with a table of practice-based insights that, according to the participants, could contribute positively to the quality of care regarding the respective theme (Tables 3-6). For readability, the term 'practitioner' is used for both GPs and MHNPs; when relevant, GP or MHNP is specified.

**Table 2.** Themes reflecting the complexity of primary mental health (MH) care provision to adults with mild intellectual disabilities (MID) from the perspective of GPs and mental health nurse practitioners (MHNPs)^a^

Theme 1: GPs’ and MHNPs’ struggles with adapting to challenging patient characteristics
Patient’s reason for encounter is often unclear or difficult to discern Multiple simultaneous problems, medical and non-medical Patients are easily disoriented when facing problems Standard guidelines for diagnosis and treatment are often not applicable to patients with MID Patients struggle with practical implementation of practitioners’ advice and organisation of care The MID often goes unrecognised or is not addressed
**Theme 2: Importance and difficulties of establishing a good doctor–patient relationship**
May be complicated by the vast difference between the lived experiences of practitioners and patient May be influenced by patient’s previous negative experiences in care or support A paternalistic stance on decision making is often adopted in the doctor–patient relationship
**Theme 3: Facilitating and hampering roles of the patient’s network**
A supporting formal or informal network can assist both practitioners and patients; however, it is not always available or lacks continuity The network can contribute to the complexity of MH problems The network may often not provide the patient with the necessary emotional and practical support
**Theme 4: GPs’ and MHNPs’ challenges to provide care in the healthcare chain**
**Finding appropriate care and support**
The practitioner’s regular network is inadequate, and there is a lack of specific networks for patients with MID There are regional differences in the organisation of care and support for patients with MID It is often unclear to practitioners how additional care and support is reimbursed It is often unclear to GPs where to request a formal IQ test and how it is reimbursed MID is often an exclusion criterion for secondary MH care Support and treatment in secondary care lack alignment with the MID Long waiting times for additional support and care
**Collaboration in the healthcare chain**
There are often multiple professionals involved around a patient, with lack of communication, coordination, and alignment between these professionals Roles and responsibilities are not clear to practitioners, other involved professionals, and patients There is lack of MID knowledge within the healthcare chain

a For readability, the term practitioner is used for both GPs and MHNPs; when relevant, GP or MHNP is specified. IQ = intelligence quotient. MH = mental health. MID = mild intellectual disabilities.

**Table 3.** Theme 1: GPs’ and MHNPs’ struggles with adapting to challenging patient characteristics

Practice-based insights that participants in our study deemed to contribute positively to the quality of primary mental health (MH) care for adults with mild intellectual disabilities (MID)^a^
**Improve knowledge, experience, and affinity with the patient group**
Improve practitioners’ confidence to rely on intuition, deviate from guidelines, and think outside the box through: More focus on this patient group in (postgraduate) GP training programmes Easily accessible consultation with a colleague practitioner or ID physician who has knowledge and experience with this patient group
**Be attentive to the patient’s need for additional support**
Within the GP practice. For example, the GP accompanying the patient to the appointment desk to schedule a follow-up appointment and ensuring that the practice’s website and practice are easily accessible for people with MID Outside the GP practice. For example, the practitioner looks up bus times with the patient, so that the patient can attend an MH service intake appointment on time
**Timely identification of MID in patients**
Improve practitioner knowledge and experience in identifying MID Develop practitioner-applicable tools for assessment and for discussing the suspicion of MID with the patient Early identification of MID by teachers, paediatricians, and public health professionals Clear information for practitioners on where to request a formal IQ test and how it can be reimbursed Registration of MID in the medical record through an episode, memo, or attention note Share knowledge of a confirmed or suspected MID with relevant parties in the healthcare chain

a For readability, the term practitioner is used for both GPs and MHNPs; when relevant, GP or MHNP is specified. ID = intellectual disability. IQ = intelligence quotient. MH = mental health. MID = mild intellectual disabilities

**Table 4.** Theme 2: Importance and difficulties of establishing a good doctor–patient relationship

Practice-based insights that participants in our study deemed to contribute positively to the quality of primary mental health (MH) care for adults with mild intellectual disabilites (MID)^a^
**Invest in the doctor–patient relationship**
The practitioner adapts to the patient’s communication level: using simple words, speaking at a slower pace, and providing frequent repetition The practitioner gives sufficient attention and time to the patient; for example, conducting longer consultations or engaging regularly in supportive consultations The practitioner provides a calm and structured environment during consultations A designated practitioner in the practice is assigned as the main point of contact and responsible for continuity of care The practitioner notes the MID and additional communication needs in the medical record so that everyone in the practice can take this into account when interacting with the patient

a For readability, the term practitioner is used for both GPs and MHNPs; when relevant, GP or MHNP is specified. MID = mild intellectual disabilities

**Table 5.** Theme 3: Facilitating and hampering roles of the patient’s network

Practice-based insights that participants in our study deemed to contribute positively to the quality of primary mental health (MH) care for adults with mild intellectual disabilities (MID)^a^
The practitioner actively involves the patient’s network in the MH trajectory The practitioner recognises the capacity of the network and provides support, coaching, and psycho-education to the network regarding the patient’s MH problems A stable network that provides long-term and consistent support to the patient, both practically and emotionally, is valuable for both the patient and the practitioner

a For readability, the term practitioner is used for both GPs and MHNPs; when relevant, GP or MHNP is specified. MH = mental health

**Table 6.** Theme 4: GPs’ and MHNPs’ challenges to provide care in the healthcare chain

Practice-based insights that participants in our study deemed to contribute positively to the quality of primary mental health (MH) care for adults with mild intellectual disabilities (MID)^a^
**Finding appropriate care and support**
A clear and up-to-date overview for practitioners of the regional network for care and support to patients with both an ID and MH problems The professionals within the network are easily accessible for the practitioner, with clear lines of communication and the possibility of personal contact Sufficient capacity and MID knowledge within support and care services, including the availability of long-term support The GPs are supported by MHNPs or professionals in the public health or social domain for the organisation and coordination of care for this patient group
**Collaboration in the healthcare chain**
The involved professionals around a patient are easily accessibility for patients, practitioners, and other professionals involved Consistency in individual care professionals involved in the patient’s care Clear communication of tasks and responsibilities with all stakeholders, including the patient Low-threshold opportunities for GPs to consult a psychiatrist, ID physician, or members of the community team A regular transfer of patient information and knowledge between involved care professionals Knowledge about implications of MID throughout the healthcare chain

a For readability, the term practitioner is used for both GPs and MHNPs; when relevant, GP or MHNP is specified. ID = intellectual disability. MH = mental health. MID = mild intellectual disabilities

### Theme 1: GPs’ and MHNPs’ struggles with adapting to challenging patient characteristics

Participants indicated that patients with both MID and MH problems often present with multiple problems simultaneously, medical and non-medical, and that these patients easily become disoriented when facing problems, manifesting in anxiety, stress, and somatic symptoms. Partly because of these aspects, this patient group frequently consults the GP.

Their reasons for encounters are often unclear or difficult to discern. Patients do not explicitly request help, have a multitude of requests, or the stated request does not align with the underlying problem:

*'They always have a lot of problems ..."What are you actually here for, what was the question?" "Oh yes", as if they completely forgot ... then it’s very difficult to go in a certain direction*.' (F3, MHNP)

Unclear encounters cause practitioners to spend a considerable amount of time clarifying the help request, untangling the multitude of problems, maintaining structure in the consultation, prioritising, and devising a plan of action.

Participants experience that this patient group generally requires a different approach for diagnosis and treatment than described in the standard MH guidelines, as patients’ abilities and circumstances often render these not directly applicable. Deviating from these guidelines and applying a suitable approach requires the practitioner to have specific knowledge and experience, which is often lacking, as stated by this participant:

*'As a young healthcare provider, you're still quite focused on yourself and cling to guidelines and protocols, but with this target group ... those don't really apply anymore, and you find yourself in a kind of swamp, and you just have to deal with it*.' (F2, GP)

Participants note that patients often appear to struggle with the practical implementation of given advice and with organising their own care. Consequently, patients often return to the GP with the same type of symptoms.

Participants consider themselves able to identify certain signs indicating (M)ID. However, they acknowledge that they frequently miss MID in patients, resulting in overburdening the patient and initiating an inadequate MH trajectory because they do not adapt their approach to the MID:

*'Those* [patients with MID] *need to be recognised to receive the appropriate care. I wonder: who is going to do that? I think it’s up to us* [GPs] *to identify them*.' (F4, GP)

Moreover, when participants do identify indicators of MID, discussing the possibility of MID with patients is found to be challenging as they do not always know how to approach it.

### Theme 2: Importance and difficulties of establishing a good doctor–patient relationship

Participants saw a good and sustainable relationship as particularly relevant for this patient group. Knowledge about the patient’s context and lived experiences can aid in assessing symptoms and patients’ follow-up of practitioner advice. Participants indicated that their necessary extra investment in building a relationship with these patients might be complicated by the vast difference between the lived experiences of a patient with MID and a practitioner, as one GP remarked:

'... *you have to understand more about how the other person is because it’s further away from you. That, I think, is really the crux, and that’s why building that relationship takes more time and also requires more time to ultimately provide good care for mental health complaints*.' (F2, GP)

Additionally, patients may have had negative experiences with care professionals in the past, challenging the establishment of a good relationship even more.

In general, participants indicated that, in the relationship with the patient, they often adopted a paternalistic stance regarding decision making, while being aware that this limited the patient’s choices. They found it difficult to assess the extent to which the patient could take responsibility in this regard and to determine their role as practitioner in this process:

*'It’s also a difficult balancing act. I find it complicated, how much control do you give to the other person, when sometimes you just want to say, "we're going to do this and this and this now because I see it’s necessary", but at the same time, you don't want to completely override someone*.' (F4, GP)

### Theme 3: Facilitating and hampering roles of the patient’s network

Some patients with MID have a formal and/or informal network that can provide support to both the practitioner and the patient. A supportive network provides the practitioner with valuable knowledge about the patient and contributes to stability in their lives in both the short and the long term. However, in the participant’s experience, networks can also be part of, or contribute to, patients' problems, by amplifying patients’ stress, underestimating or overestimating their symptoms, lacking sufficient knowledge or skills to provide appropriate support, or having unrealistic or unspoken expectations of the practitioner:

*'And especially I think not getting caught up in the patient’s panic* [by carers]*, because that’s often what I see. The patient notices that too, and then you enter a kind of circle ... those problems worsen*.' (F2, GP)

The absence of a network, or changes in individuals within the network, can create instability for the patient and affect MH problems negatively. Additionally, participants felt that the network did not always prioritise or have time for emotional support, whereas this group would benefit from that. Participants observed that, despite the presence of a network, patients continued to rely on practitioners for supportive consultations and that patients did not always feel heard by their network.

*'What you often see is that, for them* [professional carers]*, it’s more like, "I help a bit with finances and administration, and I go to the supermarket with them", but it stops there, and then you're not addressing that aspect of MH support that this group specifically needs*.' (F4, GP)

Participants saw cooperation with the network as valuable; however, it often requires extra time and effort for them to involve and inform people in the network, which is time that is not always available:

*'What I always keep in mind is how important it is to involve the network, whether it’s the personal network or the professional network, that it truly acts as a link. If you have a good understanding of that and can establish it well, I think this patient group will also rely much less on the GP*.' (F4, GP)

### Theme 4: GPs’ and MHNPs’ challenges to provide care in the healthcare chain

Participants emphasised that, in their practitioner role, they might not always be able to provide the care and support needed by patients with both MID and MH problems, especially when the problems were non-medical in nature or required expertise in MH care. Participants were convinced that timely access to appropriate support and care could limit MH problems and reduce the high demand for services in primary care practices. One participant felt isolated in providing care:

*'Then I'm treating, and I think: I'm here on my own again, but there should be much more happening in the home situation*.' (F4, GP)

#### Finding appropriate care and support

Practitioners felt responsible for organising the necessary care and support for patients, as patients or their network might not be able to do so independently. This additional organisation, coordination, and mediation of care and support required extra effort and time. Challenges mentioned included the participant’s regular network being insufficient, lack of a regional specialised network for this patient group, or frequent changes within this network, and regional differences in the organisation of care. Especially regarding context-related problems where solutions lie outside the medical domain, the participants were often not aware how and where the appropriate support was available for their patients. In addition, issues and uncertainties regarding reimbursement could hinder the organisation of care and support:

*'Everyone is also concerned about whether it fits into that box, whether there’s adequate reimbursement*.' (F3, MHNP)

Participants often experienced confusion about where to request a formal IQ test and how it should be funded. A low IQ, or the absence of an IQ test, could be an exclusion criterion for specialised care, potentially denying patients access to treatments and expertise from which they could benefit. Additionally, available care or support did not always adequately align with the needs of individuals with MID, leading to patients being referred back to the GP if they could not articulate their needs or did not benefit sufficiently from the treatment provided:

*'And they* [MH services] *have a limit for the IQ, and if they say, "you have an IQ below 75, goodbye, we're not going to start with that, figure it out yourself*."' (F2, GP)

Waiting times for MH services are often long, and considerable bureaucracy in referrals is reported. For these reasons, participants felt that sometimes the maximum achievable outcome was to continue monitoring patients themselves, without the possibility of involving external professionals.

#### Collaboration in the healthcare chain

Often, multiple professionals are involved in the care for this patient group. Regularly, there is a lack of coordination among these providers, as patients and their network may not be able to fulfil this role. Practitioners in this study often assumed the coordination role but faced challenges, as other care professionals might not be easily accessible, changed frequently, lacked adequate feedback mechanisms, and might be uncertain about roles and responsibilities. Lack of knowledge about MID among professionals involved was also an issue. When an MH trajectory is not functioning as intended, the GP is often the first point of contact for both the patient and other involved professionals. This may lead to feelings of irritation, as experienced by this participant:

*'If there’s any irritation on my part, it’s indeed more often in communication with other care professionals than with the client ... there’s still often a feeling of separation where you're trying to toss something over instead of actively working together*.' (F2, GP)

## Discussion

### Summary

In this study, we explored GPs’ and MHNPs’ experiences, needs, and recommendations for improvement regarding primary MH care. Participants perceived their patients with both MID and MH problems as complex cases. This related to patient factors, challenges experienced in the doctor–patient relationship, the presence and capacity of the patient’s network, and orchestrating care in the healthcare chain. As a result, participants found it difficult to provide the appropriate care and that patients relied substantially on their GP, even for problems of a non-medical nature. These difficulties were often experienced as a burden. Practitioners believed that, if this patient group received appropriate care and support from the (in)formal network, practitioners, and other care professionals, MH problems might be limited at an early stage, the work pressure on primary care practices would decrease, and more intensive forms of MH care could, in some cases, be prevented.

### Strengths and limitations

This study has several strengths. First, the focus groups were guided by a topic guide based on four themes that emerged from a previous interview study among patients with MID who received primary MH care. This topic guide was critically scrutinised by both the research team and the study’s advisory group to ensure that practitioners’ perspectives were collected as completely and broadly as possible. By integrating the perspectives of those who received and those who provided the care, valuable information was retrieved to inform research, practice, and policy about opportunities to improve primary MH care for people with MID. Second, participants had varying degrees of experience with the patient group and were reasonably equally distributed across The Netherlands. Finally, the focus groups were moderated by a trained researcher who, as an ID physician, could easily relate to the participants’ clinical experiences, thereby adding to the depth of the discussion.

The main limitation of this study is that the practitioners’ perspectives may be partly determined by the Dutch primary care model, reducing external validity. However, various countries have comparative primary care models, rendering the suggested implications for research and practice also valuable and applicable outside The Netherlands. Furthermore, in countries with weaker formal primary care systems, professionals working in non-institutionalised settings with people with both MID and MH will experience many of the problems described here.

### Comparison with existing literature

Our findings show some similarities with previous qualitative studies focusing on people with MH problems and a wider range of ID than MID.^1,15^ However, in contrast to our study, concrete suggestions to improve care were scarcely provided in those studies. Only one study of which we are aware focuses on GPs’ views on primary MH care for people with ID.^1^ In that interview study, GPs also experienced problems in referring patients, and, as in our study, they described knowledge of the patients’ background, continuity in the GP–patient relationship, and interdisciplinary meetings as helpful in providing optimal care.

Our results also resemble those of a systematic qualitative review study that focused on general MH care for people with ID by a broad range of MH care professionals, including GPs, psychiatrists, psychologists, therapists, and nurses.^15^ That study also described the complexity of patient problem presentation and high service demands, the extensive carer resources and time required, and the emotional effects (for example, feeling alone) on care professionals.

### Implications for practice

Several opportunities that may improve care have been revealed. First, recognising and establishing MID in patients, and taking into account the MID throughout the MH trajectory, is of utmost importance.^16^ This is preferably done as early as possible in a person’s life and not solely as a task of a GP, but also of teachers, paediatricians, and social care professionals in the public health domain. This means that it is important to invest in training programmes on MID recognition not only for GPs, but also for other professionals who may encounter this patient group. Those training programmes should focus on screening tools for MID, for example the Screener for Intelligence and Learning Disabilities (SCIL) and Hayes Ability Screening Index (HASI),^17,18^ and also on conversational techniques on how to discuss a suspected MID with a patient. When a formal IQ test is required, this test should be easily accessible, with clarity about local test locations and financing.

Second, on the GP-practice organisational level, the following two aspects will contribute to a good, sustainable doctor–patient relationship: (1) optimal continuity in care provided in the practice by the same person;^19^ (2) registering the MID and additional communication needs in the patient’s file. This can act as a reminder for the practitioner’s assistant to schedule extra time for a consultation, thus allowing the practitioner to give sufficient attention and time to the patient during consultations and adapt the level of communication to the MID. From an earlier study, it appears that more than 80% of people with MID were not registered as such in Dutch primary care.^5^ In the UK, registration is higher thanks to the register for people with ID developed for the Quality and Outcomes Framework, an incentive programme for all GP practices in the UK.^20^ However, there too, there are people with MID who are not registered as such in primary care.^21^

Third, practitioners need to actively involve the patient’s network. Regarding the informal network, it is important for practitioners to be aware of the network’s capacity and identify if additional support for the network or patient is needed.^22^ A strong formal network consists of familiar professionals who are able to provide both practical and emotional support to people with MID, for whom it is clear what their practitioners and carers expect of them.^23^ Therefore, it is important that these professionals have adequate training and peer supervision opportunities, with clear tasks and an appropriate workload.^24^

Fourth, because patients with both MID and MH problems often present with multiple problems that are often not within the practitioner’s scope, the practitioner must have access to an adequate regional network of both medical and non-medical professionals who work closely and in coordination. The visibility and accessibility of these professionals are not always evident for Dutch practitioners. In contrast to the UK and the US, in The Netherlands there are no ID psychiatrists, community learning disability teams, or ID nurses available to support practitioners in the care for this patient group.^25–27^ These professionals play a role in coordinating care, improving individual's physical and mental health, reducing barriers to independent living, supporting individuals in leading a fulfilling life, and advocating strongly for improved knowledge about the care for patients with ID in general. The experiences gained internationally can provide insights into whether and how the introduction of an ID nurse would be of added value in Dutch primary care practices.

Finally, interprofessional collaboration is more effective when there is adequate reimbursement, a team vision, shared goals, formal quality processes, and shared information systems.^28,29^

In conclusion, practitioners perceive patients with both MID and MH problems as complex patients who present with multiple problems simultaneously, medical and non-medical, and continue to rely on them, even if the problems are non-medical in nature. Consequently, practitioners often experience providing care and support to this patient group as burdensome. In order to provide effective primary MH care, it is important for practitioners and the other professionals involved to invest in a strong relationship with the patient, consider MID throughout the MH trajectory, and establish a stable and sustainable support network and coordinated collaborative care around the patient. Organising effective care and support may improve the quality of primary MH care, decrease the pressure on primary care practices, and, in some cases, prevent more intensive forms of MH care.

## Data

The dataset relied on in this article is available from the corresponding author on reasonable request.
